# Low Complexity Estimation Method of Rényi Entropy for Ergodic Sources †

**DOI:** 10.3390/e20090657

**Published:** 2018-08-31

**Authors:** Young-Sik Kim

**Affiliations:** Department of Information and Communication Engineering, Chosun University, 309 Pilmoondae-ro Dong-gu, Gwangju 61452, Korea; iamyskim@chosun.ac.kr; Tel.: +82-62-230-7032

**Keywords:** entropy estimation, Shannon entropy, Rényi entropy, quadratic entropy, random number generation, nearest neighbor distance, security

## Abstract

Since the entropy is a popular randomness measure, there are many studies for the estimation of entropies for given random samples. In this paper, we propose an estimation method of the Rényi entropy of order α. Since the Rényi entropy of order α is a generalized entropy measure including the Shannon entropy as a special case, the proposed estimation method for Rényi entropy can detect any significant deviation of an ergodic stationary random source’s output. It is shown that the expected test value of the proposed scheme is equivalent to the Rényi entropy of order α. After deriving a general representation of parameters of the proposed estimator, we discuss on the particular orders of Rényi entropy such as α→1, α=1/2, and α=2. Because the Rényi entropy of order 2 is the most popular one, we present an iterative estimation method for the application with stringent resource restrictions.

## 1. Introduction

Since the entropy is a popular randomness measure, many studies are devoted to the efficient estimation of the Shannon or Rényi entropy for given random samples. In particular, one of the important applications for entropy estimator is random number generators (RNGs). RNG is one of the fundamental cryptographic primitives and a good RNG can be modelled as an ergodic random source. For block ciphers and public key cryptography, an RNG also can be used as a key-stream generator. In addition, the digital signature algorithm (DSA) requires a random number for its computation [1]. Since a statistical bias in random numbers can be exploited to reduce the computational complexity of the exhaustive search by an attacker, the entire security of the crypto-systems usually depends on the statistically quality of RNG output.

In order to obtain the unpredictability of random output, many crypto-systems require a true (physical) random number generator (TRNG) [2,3,4] as well as pseudo-random number generators (PRNG). However, a TRNG output can be easily influenced by environments such as temperature, electro-magnetic wave, and so on. Therefore, an on-the-fly statistical test scheme, as known as online test, is requested to guarantee the statistical quality of RNG output in cryptographic standards [5]. In particular for the applications with stringent resource constraints such as sensor nodes, smart cards, etc., an online test scheme for RNG should both have compact size in hardware/software and detect a various range of statistical bias to ensure the security of the systems [6]. That is, we need a good and efficient test scheme of the statistical quality of random sources. Therefore, it is highly desirable to construct low-cost and reliable entropy estimation methods of the random output of TRNG.

When it comes to the randomness measure, the Shannon entropy is one of the widely used measures. Let $F_{2}=\left\{0,1\right\}$ be the finite field with two elements. Let *X* be a random variable for *L*-bit random symbols in $F_{2}^{L}$ from the random source *S* and the probability of occurring random symbol *b* from the random source *S* is denoted by Pr(b). Then, the Shannon entropy of *L*-bit blocks from the random source *S* is defined as

(1) $$\begin{array}{c} H_{L}\left(X\right)=-\sum_{b\in F_{2}^{L}}\mathrm{Pr}\left(b\right)\log_{2}\mathrm{Pr}\left(b\right). \end{array}$$

In various literature, there have been developed efficient estimators of the Shannon entropy [7,8,9,10,11]. In addition, the complexity of estimating the Shannon entropy of a distribution on *k* elements from independent samples also has been developed [10,12,13].

In 1961, another generalized entropy measure is defined by Rényi [14]. The Rényi entropy is popularly used in a number of signal processing and pattern recognition applications [15,16,17]. For example, Rényi entropy has been used in cryptography, in the study of bio-informatics, and in the bio-medical applications [18]. Sometimes, the Rényi entropy can provide more strict randomness measure for the cryptographic applications such as the privacy amplification [19]. Rényi entropy of order α for *L*-bit blocks from the random source *S* is defined as

(2) $$\begin{array}{c} R_{\alpha ,L}\left(X\right)=\frac{1}{1-\alpha }\log_{2}\sum_{b\in F_{2}^{L}}\mathrm{Pr}{\left(b\right)}^{\alpha }, \end{array}$$

where α>0 and α≠1. Note that Rényi entropy is defined as the log base 2 of the expectation of $\mathrm{Pr}{\left(b\right)}^{\alpha -1}$ normalized by 1−α.

Generally, a random source used in a cryptographic protocol should have a maximum entropy. As a result, Shannon entropy (or Rényi entropy) is recognized as one important measure of randomness. For example, standards such as NIST STS (Statistical Test Suits) [20] or AIS.31 [5], a widely used standard for evaluating TRNGs, include Entropy Estimation items. The proposed method estimates the actual Rényi entropy value very accurately, as can be confirmed from the simulation results. Therefore, if the estimation result shows a lower value (from the maximum), it can be interpreted as a signal that the random sources are generating a significant deviated output from the perfect.

The Rényi entropy is a generalization of the Shannon entropy since it contains the definition of the Shannon entropy when α approaches to one [21]. In particular, notice that it is easy to prove the Rényi entropy is always less than or equal to Shannon entropy by using Jensen’s inequality [22] for α>1, i.e.,

(3) $$\begin{array}{c} R_{\alpha ,L}\left(X\right)\leq H_{L}\left(X\right). \end{array}$$

Here, the equality holds for the equiprobable random source. Therefore, Rényi entropy can be used as the lower bound of the Shannon entropy for a random source *S*.

There are some studies for the estimation of Rényi entropy [15,23,24]. In particular, many applications such as machine learning [16], blind deconvolution of linear channels [25], information flows in financial data [26] and cryptography [19] have paid attention to the Rényi entropy of order 2, also called the *quadratic entropy* or *collision entropy* [15]. The most straightforward approach for entropy estimation is the direct calculation of entropy based on the probability mass function (pmf) or the probability density function (pdf) of empirical data. For example, Erdogmus and Principe proposed the Rényi entropy or order estimator by using the non-parametic estimation of the pdf of a random variable [16]. In order to estimate the pdf of a given sample distribution, they used the Parzen windowing method, in which the pdf is approximated by the sum of kernels such as the Gaussian function. In addition, there are many non-parametric approaches to estimate entropies, which are usually based on the data compression [27] or the nearest neighbor distance [7,8,28,29]. There are results on the estimation of Rényi entropy rate of Markov chains [30]. This can be also considered for randomness measure for RNG because a skewed RNG can be modelled as Markov chain with vaying transition probability. However, many of them are not suitable in constrained devices with stringent resource restriction due to the computational complexity.

In this paper, we propose a low complexity estimation method of the Rényi entropy of order α, where α is a real number. This method does not require any initialization phase contrary to the previous Maurer’s universal statistical test [8] and Coron’s refined test [7] which are widely used for estimating Shannon entropy especially in cryptographic applications [5,20]. In the proposed scheme, we can estimate the Rényi entropy of order α without introducing complicated computations such as logarithms or divisions. We show that the expected value of the proposed test function is equivalent with the Rényi entropy of order α. That is, the output of the proposed estimation method almost surely converges to the Rényi entropy of order α values with large samples. Because the requirement of a large sample size for accurate estimation can be a drawback of the proposed scheme in some applications, we also propose an iterative algorithm for the Rényi entropy of order 2, which requires a relatively short sample size and shows accurate test results. Using the simple counting method, we can efficiently implement test module of RNGs based on the Rényi entropy of order 2. Therefore, it can be used as a statistical tester of RNGs for many embedded security systems such as smart cards. This is an extended version of the conference paper [31]. In this paper, we generalized the order from integer to real number. Also, iterative estimation method is included for more constrained environments.

The remainder of this paper is organized as follows: in Section 2, previous entropy estimation schemes are reviewed. In particular, for the comparison, Maurer’s universal statistical test and Coron’s refined scheme are presented in detail since they are the popular nearest neighbor distance based schemes, which are exploited by the proposed scheme. In Section 3, the proposed estimation scheme for the Rényi entropy of order α is presented. Firstly, we describe the estimation method and show that the expected value of the proposed test function is equivalent with the Rényi entropy of order α. We suggest the iterative estimation algorithm, which requires a relatively small sample size. Then, numerical results are given in Section 4. For the three block sizes and two sample sizes, the estimated values are compared with the Rényi entropy of order 2 or order 1/2. For the iterative algorithm, we can check that the proposed algorithm is more stable with less sample size. Finally, we conclude this paper in Section 5.

## 2. Previous Works

### 2.1. The Nearest Neighbor Distance

In 1992, Maurer proposed the universal statistical test for evaluating statistical quality of random number generators [8]. By universal, this method can detect various kinds of statistical defects in random data. Maurer conjectured that the test result of his estimation method is related to Shannon entropy of *L*-bit blocks. Later, this conjecture was proved by Coron and Naccache [32]. The Maurer’s universal statistical test is included in the statistical test suite by NIST for evaluating RNGs in cryptographic applications [20].

Let N=(Q+K)L. Let $s^{N}$ denote the generated random sequence with length *N*. In the Maurer’s universal statistical test, an initialization phase is required for the first *Q* *L*-bit blocks. In order to make it so each of $2^{L}$ blocks occurs at least once during the initialization phase with high probability, the size of *Q* should be greater than $10\times 2^{L}$ [8]. Then, in the evaluation phase, the next *K* *L*-bit blocks are used for the entropy estimation.

Let $b_{n}\left(s^{N}\right)=\left[s_{L(n-1)+1},\cdots ,s_{Ln}\right]$ be the *n*-th *L*-bit block of $s^{N}$. Then, the Maurer’s universal statistical test is based on the following test function:

$$\begin{array}{c} f_{M}\left(s^{N}\right)=\frac{1}{K}\sum_{n=Q+1}^{K+Q}\log_{2}D_{n}\left(s^{N}\right), \end{array}$$

where

(4) $$\begin{array}{c} D_{n}\left(s^{N}\right)=\left\{\begin{array}{cc} n, & \mathrm{if}\;\forall i<n,b_{n}\left(s^{N}\right)\neq b_{n-i}\left(s^{N}\right),\\ \min\{i\;|\;i\geq 1,b_{n}\left(s^{N}\right)=b_{n-i}\left(s^{N}\right)\}, & \mathrm{otherwise}. \end{array}\right. \end{array}$$

That is, $D_{n}\left(s^{N}\right)$ is a distance between the index of the current pattern *b* and the nearest previous index of the same pattern *b*. Note that this distance $D_{n}\left(s^{N}\right)$ is conversely proportional to the probability of occurring the pattern *b*. That is, if the probability of occurring the pattern *b* is small, the expected value of the distance $D_{n}\left(s^{N}\right)$ is large, and vice versa. In fact, Coron and Naccache proved that Maurer’s universal statistical test is closely related to the Shannon entropy for a source emitting the sequence of binary random variables, $U^{N}=U_{1},\cdots ,U_{N}$ as follows [7]:

(5) $$\begin{array}{c} \lim_{L\to \infty }\left[E\left[f_{M}\left(U^{N}\right)\right]-H_{L}\left(U^{N}\right)\right]=\int_{0}^{\infty }e^{\zeta }\log_{2}\zeta d\zeta ≅-0.8327462. \end{array}$$

Later, Coron refined the Maurer’s universal statistical test as an exact entropy estimator without the numerical discrepancy presented in (5) [7].

Therefore, Coron’s refined test is adopted as an estimating method of Shannon entropy in AIS.31 specification, the German standard for cryptographic TRNGs [5]. Coron modified test function as

(6) $$\begin{array}{c} f_{C}\left(s^{N}\right)=\frac{1}{K}\sum_{n=Q+1}^{K+Q}g\left(D_{n}\left(s^{N}\right)\right), \end{array}$$

where

(7) $$\begin{array}{c} g\left(i\right)=\frac{1}{\ln(2)}\sum_{k=1}^{i-1}\frac{1}{k} \end{array}$$

and *K* and *Q* are given as the same parameters of the Maurer’s universal statistical test. Then, he proved that the expected value of the test function $f_{C}\left(s^{N}\right)$ is equal to the Shannon entropy of *L*-bit blocks of the random source as follows [7]:

$$\begin{array}{c} E\left[f_{C}\left(U^{N}\right)\right]=H_{L}\left(U^{N}\right). \end{array}$$

Note that, in Coron’s test, a logarithm is substituted by a summation of a series of integer divisions. In his paper, he proposed the approximated method in order to reduce computational complexity as follows [7]:

(8) $$\begin{array}{c} \sum_{k=1}^{i-1}\frac{1}{k}\approx \frac{1}{\ln(2)}\ln\left(i-1\right)+\frac{1}{2(i-1)}-\frac{1}{12{\left(i-1\right)}^{2}}+O\left(\frac{1}{{\left(i-1\right)}^{4}}\right)-0.577216. \end{array}$$

### 2.2. Previous Entropy Estimation Approach

One of the widely used estimators for Shannon or Rényi entropy is the “plug-in” estimator. The “plug-in” approach estimates parameters and then substitutes them into the entropy function, (1) or (2). For example, let $N_{k}$ be the frequency of the symbol *k* and *N* the total number of samples. Then, we have probability mass function (pmf) $p_{k}=N_{k}/N$. Using this pmf, we can directly calculate entropy of the given sample using (1) or (2).

Since the pdf of a random sample is unknown a priori, the estimation of pdf of a random variable is complicated and usually contains some complex functions. For example, Erdogmus and Principe proposed the estimation of entropy based on the non-parametic direct estimation of pdf, that is, the Parzen window method in the context of minimizing error entropy [16]. The Parzen estimator of the error pdf $f_{e}\left(\zeta \right)$ is given by

(9) $$\begin{array}{c} {\hat{f}}_{e}\left(\zeta \right)=\frac{1}{N}\sum_{i=1}^{N}\kappa \left(\zeta -e_{i},\sigma^{2}\right), \end{array}$$

where *N* is the sample size and κ is the kernel function, usually implemented by using the multidimensional Gaussian function with a radially symmetric variance $\sigma^{2}$. Then, we can directly calculate the Rényi entropy or Shannon entropy using the estimated pdf in (9). However, the computation of the sum of the Gaussian functions is usually infeasible in most constrained devices.

## 3. New Estimation Method of the Rényi Entropy of Order α

In this section, we derive the parameters for the estimation of the Rényi entropy of order α, for a real number α. In this derivation, we assume that an ergodic random source *S* and random sequences from the source *S* are over $F_{2}$ and consecutive and distinct *L* symbols are treated as a basic element of test function. Thus, the maximum value of Rényi entropy (and also Shannon entropy) will be *L*-bit. For the estimation of Rényi entropy, we firstly focus on the estimation of inner summation in (2). Then, to obtain exact value of Rényi entropy, the logarithm and division by 1−α will be applied to the result of the estimation. The test function is given as

(10) $$\begin{array}{c} f\left(s^{N}\right)=\frac{1}{K}\sum_{n=1}^{K}g\left(D_{n}\left(s^{N}\right)\right), \end{array}$$

where $D_{n}\left(s^{N}\right)$ is the index distance defined in (4). Now, for given real number α and the index distance $D_{n}\left(s^{N}\right)=k$, we are going to find the values of g(k) for each k≥1 which is closely related to the inner summation (2) for the estimation of Rényi entropy of order α. The following theorem gives us the general representation of g(k) for given α.

**Main Result: Proposed Test Function of Rényi Entropy of Order α**

*For the estimation of Rényi entropy of order α, the parameters g(k) of estimator for given index distance k in (10) are given as*

(11) $$\begin{array}{c} g\left(k\right)=\left\{\begin{array}{cc} 1, & if\;k=1,\\ {\left(-1\right)}^{k-1}P_{k-1}^{\alpha -2}, & if\;k\geq 2, \end{array}\right. \end{array}$$

*where*

(12) Pk−1α−2=α−2k−1=(α−2)(α−3)⋯(α−k)(k−1)!.

*The derivation of the proposed test function can be justifed as in the following proof.*

**Proof.**  We start from the the expectation of the test function $f(s^{N})$ given as

(13) $$\begin{array}{c} E\left[f\left(U_{S}^{N}\right)\right]=\sum_{k=1}^{\infty }\mathrm{Pr}\left[D_{n}\left(U_{S}^{N}\right)=k\right]g\left(k\right), \end{array}$$

where $U_{S}^{N}$ is a vector of random variables for random sequence $s^{N}$ of *L*-bit symbols and g(k) is the *k*-th parameter for the estimation. Then, the probability $\mathrm{Pr}[D_{n}\left(U_{S}^{N}\right)=k]$ can be represented as

$$\begin{array}{c} \mathrm{Pr}\left[D_{n}\left(U_{S}^{N}\right)=k\right]=\sum_{b\in B^{L}}\mathrm{Pr}\left[b_{n}=b,b_{n-1}\neq b,\cdots ,b_{n-k+1}\neq b,b_{n-k}=b\right]. \end{array}$$

If the random variable is stationary, we have

(14) $$\begin{array}{c} \mathrm{Pr}\left[D_{n}\left(U_{S}^{N}\right)=k\right]=\sum_{b\in B^{L}}\mathrm{Pr}{\left[b\right]}^{2}{\left(1-\mathrm{Pr}\left[b\right]\right)}^{k-1}. \end{array}$$

From (13) and (14), the expectation can be represented as

$$\begin{array}{c} E\left[f\left(U_{S}^{N}\right)\right]=\sum_{b\in B^{L}}\mathrm{Pr}\left[b\right]\gamma \left(\mathrm{Pr}\left[b\right]\right), \end{array}$$

where γ(·) is defined as

$$\begin{array}{c} \gamma \left(x\right)=x\sum_{k=1}^{\infty }{\left(1-x\right)}^{k-1}g\left(k\right). \end{array}$$

Here, we are going to find the representation of g(k) that satisfies the expected value of $E\left(f\left(U_{S}^{N}\right)\right)=\sum_{b\in F_{2}^{L}}\mathrm{Pr}{\left(b\right)}^{\alpha }$. Then, we have

$$\begin{array}{c} \gamma \left(x\right)=x\sum_{k=1}^{\infty }{\left(1-x\right)}^{k-1}g\left(k\right)=x^{\alpha -1}. \end{array}$$

By removing *x* at both sides, the equation is simplified as

$$\begin{array}{c} \sum_{k=1}^{\infty }{\left(1-x\right)}^{k-1}g\left(k\right)=x^{\alpha -2}. \end{array}$$

By substituting x=1−t, we have

(15) $$\begin{array}{c} \sum_{k=1}^{\infty }t^{k-1}g\left(k\right)={\left(1-t\right)}^{\alpha -2}. \end{array}$$

From (15), for α=2, we have $\sum_{k=1}^{\infty }t^{k-1}g\left(k\right)=1$. That is, g(1)=1, otherwise g(k)=0. For α≠2, the Tayler series at t=0 of the right hand side of (15), ${\left(1-t\right)}^{\alpha -2}$, is given as

(16) (1−t)α−2=∑k=0∞α−2ktk(−1)k,

where

α−2k=Pkα−2=(α−2)(α−3)⋯(α−1−k)k!

and $P_{0}^{\alpha -2}=1$. Note that the combination in (16) is a generalized binomial expansion for the real number α and a positive integer *k* [33]. Thus, we have

$$\begin{array}{c} \sum_{k=0}^{\infty }t^{k}g\left(k+1\right)=\sum_{k=0}^{\infty }{\left(-1\right)}^{k}P_{k}^{\alpha -2}t^{k}. \end{array}$$

Finally, the parameter g(k) of the estimator for the exact Rényi entropy of order α for a real number α is given as

$$\begin{array}{c} g\left(k\right)=\left\{\begin{array}{cc} 1, & \mathrm{if}\;k=1,\\ {\left(-1\right)}^{k-1}P_{k-1}^{\alpha -2}, & \mathrm{if}\;k\geq 2. \end{array}\right. \end{array}$$

 ☐

Table 1 shows examples of parameters of the proposed estimator for some cases of α. For the integer α≥3, we can see the negative values of g(k). This means that the test function in (10) may be negative after accumulation of parameters for given random samples. Therefore, in this case, we need to take absolute value of test result before applying logarithm to calculate actual Rényi entropy of order α.

Now we are going to derive representation of g(k) for some particular orders, α→1, α=1/2, and α=2 in the next subsections. First, let us start from the case of α approaching to 1, where Rényi entropy converges to Shannon entropy.

### 3.1. Convergence of Rényi Entropy and Shannon Entropy

The proposed estimation method converges to the same estimator of Shannon entropy by Coron [7] as in the following theorem.

**Theorem** **1.** *The proposed test function of Rényi entropy converges to the test function of Shannon entropy by Coron when α goes to 1.*

**Proof.**  If α→1, from (11), the parameter converges

(17) $$\begin{array}{cc} g(k) & \to {\left(-1\right)}^{k-1}\frac{\left(-1)(-2)\cdots (-(k-1)\right)}{(k-1)!}=1. \end{array}$$

This means that every case will be counted and the test function $f(s^{N})$ in (10) will always converge to 1. To obtain actual value of Rényi entropy, the test function $f(s^{N})$ should be applied by the logarithm of base 2 and divided by (1−α)→0. To obtain the converged value for α→1, we can use L’Hospital’s theorem to $\frac{\log_{2}f\left(s^{N}\right)}{1-\alpha }$. Then, we have

(18) $$\begin{array}{cc} \lim_{\alpha \to 1}\frac{\log_{2}f\left(s^{N}\right)}{1-\alpha } & =\lim_{\alpha \to 1}\frac{d(\log_{2}f\left(s^{N}\right))}{d\alpha }\frac{1}{\frac{d(1-\alpha )}{d\alpha }}\\ & =\lim_{\alpha \to 1}-\frac{1}{f(s^{N})\ln2}\frac{df(s^{N})}{d\alpha }\\ & =\lim_{\alpha \to 1}-\frac{1}{f(s^{N})\ln2}\frac{1}{K}\sum_{n=1}^{K}\frac{dg(D_{n}\left(s^{N}\right))}{d\alpha }. \end{array}$$

From (11), we can obtain the derivative of g(k) with respect to α as

(19) $$\begin{array}{cc} \lim_{\alpha \to 1}\frac{dg(D_{n}\left(s^{N}\right))}{d\alpha } & ={\left(-1\right)}^{i-1}[\frac{\left(\alpha -3)\cdots (\alpha -i\right)}{(i-1)!}+\frac{\left(\alpha -2)(\alpha -4)\cdots (\alpha -i\right)}{(i-1)!}\\ & \;+\cdots +\frac{\left(\alpha -2)\cdots (\alpha -(i-1))(\alpha -i\right)}{(i-1)!}{]}_{\alpha =1}\\ & =-1-\frac{1}{2}-\frac{1}{3}-\cdots -\frac{1}{i-1}=-\sum_{k=1}^{i-1}\frac{1}{k}. \end{array}$$

From (17), we also have $f\left(s^{N}\right)=\frac{1}{K}\sum_{n=1}^{K}1=1$ when α goes 1. Therefore, from (18) and (19), we have

$$\begin{array}{cc} \lim_{\alpha \to 1}\frac{\log_{2}f\left(s^{N}\right)}{1-\alpha } & =\frac{1}{K}\sum_{n=1}^{K}G\left(D_{n}\left(s^{N}\right)\right), \end{array}$$

where $G\left(D_{n}\left(s^{N}\right)\right)=\frac{1}{\ln2}\sum_{k=1}^{i-1}\frac{1}{k}$ for $D_{n}\left(s^{N}\right)=i$. It is exactly the same result by Coron given in (6) and (7) [32]. ☐

That is, the proposed estimator includes the previous result as a special case and it can be considered as evidence that the proposed approach is valid for the entropy estimation.

### 3.2. Proposed Test Function for Rényi Entropy of Order $\frac{1}{2}$

In this subsection, we will derive a simplified test function for Rényi entropy of order 1/2. This order of Rényi entropy is closely related to the exponent of the average growth rate of average guesswork [34,35]. From the main result, for k>1 and $\alpha =\frac{1}{2}$, we have

(20) g(k)=(−1)k−1−32k−1=(−1)k−1(−32)(−52)⋯(−2k−12)(k−1)!=(−1)2(k−1)1×3×⋯×(2k−1)2k−1(k−1)!=1×2×3×4×⋯×(2k−2)(2k−1)22(k−1)[(k−1)!]!=(2k−1)!4k−1[(k−1)!].

However, the calculation of the factorial is a complicated task and takes a long time even for the moderate size of integer. Therefore, we need to simplify (20) for the practical implementation. First, we can use the Stirling’s approximation given as

$$\begin{array}{c} k!\approx k^{k}e^{-k}\sqrt{2\pi k}. \end{array}$$

Then, we have

$$\begin{array}{cc} \frac{(2k-1)!}{4^{k-1}{\left[\left(k-1\right)!\right]}^{2}} & =\frac{\sqrt{2\pi }\times {\left(2k-1\right)}^{2k-1}e^{-(2k-1)}\sqrt{2k-1}}{4^{k-1}\times 2\pi \times {\left(k-1\right)}^{2(k-1)}e^{-2(k-1)}\left(k-1\right)}\\ & =\frac{\sqrt{2k-1}}{e\sqrt{2\pi }\times 4^{k-1}}\times {\left(\frac{2k-1}{k-1}\right)}^{2k-1}\\ & =\frac{2\sqrt{2k-1}}{e\sqrt{2\pi }}\times {\left(\frac{2k-1}{2k-2}\right)}^{2k-1}\\ & =\frac{2\sqrt{2k-1}}{e\sqrt{2\pi }}\times {\left(1+\frac{1}{2(k-1)}\right)}^{2k-1}. \end{array}$$

Note that, for large enough *k*, the right-most factor in the last equality converges to the natural number *e* as follows:

$$\begin{array}{c} \lim_{k\to \infty }{\left(1+\frac{1}{2(k-1)}\right)}^{2k-1}\approx \lim_{k\to \infty }{\left(1+\frac{1}{k}\right)}^{k}=e. \end{array}$$

Thus, we have for large *k*

(21) $$\begin{array}{c} g\left(k\right)=\frac{(2k-1)!}{4^{k-1}{\left[\left(k-1\right)!\right]}^{2}}\approx \frac{2e\sqrt{2k-1}}{e\sqrt{2\pi }}=\sqrt{\frac{2}{\pi }\left(2k-1\right)}. \end{array}$$

When it comes to a big enough size of *k*, if k≥10, the error rate of the original value of g(k) and its approximation in (21) is less than 1.31%. In Section 4, we use only the first five g(k) for Rényi entropy of order 1/2 such as g(1)=1, g(2)=1.5, g(3)=1.875, g(4)=2.1875, and g(5)=2.4609. The remainder terms are estimated as $\sqrt{\frac{2}{\pi }\left(2n-1\right)}=0.7979\sqrt{2n-1}$ for n≥6:

$$\begin{array}{cc} f(s^{N}) & =\frac{1}{K}\sum_{n=1}^{K}g\left(D_{n}\left(s^{N}\right)\right)\\ & =\frac{1}{K}\sum_{n=1}^{5}A_{n}g\left(n\right)+\frac{1}{K}\sum_{n=6}^{K}A_{n}\sqrt{\frac{2}{\pi }}\sqrt{2\times n-1}, \end{array}$$

where $A_{n}$ (n≥1) is the number of symbol *k* of random samples with length *N*. In Section 4, we will see the small block size such as L=4 or L=6, the number of uses of exact values of g(k) should be large to obtain more exact estimation results. However, for L=8, only the first five exact values of g(k) is enough to obtain good results.

### 3.3. Estimation of Collision Entropy

In this section, we discuss the estimation method of the Rényi entropy of α=2 for *L*-bit blocks. This case is both one of the widely used Rényi entropy orders and we can very efficiently implement the estimator for this case. We will see that this case is based on a simple counting of consecutive occurrence of the same *L*-bit random samples. The test value eventually converges to the Rényi entropy of order 2 with increasing sample size.

Assume an ergodic random source *S*. Then, from (12), the test function for ‘collision entropy’ is given as

(22) $$\begin{array}{c} f_{R}\left(s^{K}\right)=-\log_{2}\frac{1}{K}\sum_{n=1}^{K}g\left(D_{n}\left(s^{K}\right)\right), \end{array}$$

where

(23) $$\begin{array}{c} g\left(k\right)=\left\{\begin{array}{cc} 1, & \mathrm{if}\;k=1,\\ 0, & \mathrm{otherwise}. \end{array}\right. \end{array}$$

The proposed scheme can be classified as the entropy estimator based on the nearest neighbor distance, which is also used in the Maurer’s and Coron’s tests.

From the main result, it can be readily proved that the expected value of the proposed test function in (22) is equivalent to the Rényi entropy of order 2 as in the following proposition.

**Proposition** **1.** *The expected value of the test function in (22) is equivalent with the Rényi entropy of order 2 (collision entropy) of L-bit sample from an ergodic random source S.*

Notice that the test function in (22) can be efficiently implemented as in the following description. Let $V_{th}$ be the threshold to accept a given sample as random. The threshold $V_{th}$ can be determined according to applications and a relevant statistical significant level. For implementation, the division and log base 2 in (22) are not essential for the decision and it is enough to only count the number of occurrences such that $D_{n}\left(s^{K}\right)=1$. For example, in order to accept the given sample as random, the test function in (22) should be greater than the specified threshold of estimated entropy value as follows:

(24) $$\begin{array}{c} f_{R}\left(s^{K}\right)=\log_{2}K-\log_{2}\sum_{n=1}^{K}G_{n}\left(s^{K}\right)>V_{th}. \end{array}$$

Then, (24) can be converted into the following relation:

(25) $$\begin{array}{c} \sum_{n=1}^{K}G_{n}\left(s^{K}\right)<K\cdot 2^{-V_{th}}. \end{array}$$

Since the right-hand side (RHS) is fixed in (25) when the sample size *K* is also fixed, it is enough to check the number of times that the specified event on the left-hand side (LHS) is less than the pre-determined value in the RHS. This testing function will be referred to as the *basic test* of the iterative estimation algorithm presented in the following subsection.

Now, let us compare the computational complexity required by the proposed Rényi entropy estimation method with the complexity required by another entropy estimation methods based on the neareast neighbor distance. The required number of operations for three entropy estimation methods based on the nearest neighbor distance is listed in Table 2.

As you can seed in Table 2, the proposed method can minimize the number of logarithms and divisions for estimation. Note that the logarithm or division is much more complicated than summation. Therefore, the proposed method has the lowest computational complexity when it is compared with the other nearest neighbor distance based estimations, Maurer’s method in NIST STS [20] and Coron’s method in AIS.31 [5].

### 3.4. Iterative Estimation Algorithm for Collision Entropy

For the accurate estimation, the proposed test scheme requires a large sample size, which can be a drawback of the proposed scheme in some applications since it takes much time to collect enough samples for a single estimation. In order to mitigate this drawback, we propose an iterative testing scheme, which will always watch the generated random samples on-the-fly and continuously update the test value with a new counting result for a shorter sample size. The proposed iteration algorithm is presented in Algorithm 1.

In Algorithm 1, $N_{S}$ is the sample size for the basic test and *w* (0≤w<1) is the weight of the previously accumulated value. Algorithm 1 consists of basic tests, which are continuously carried out when the test is running. The inside statements of for-loop in Algorithm 1 correspond to the basic test that is explained in Section 3.3. Let $N_{I}$ be the number of iterations. Algorithm 1 can be justified as in the following proposition.

**Algorithm 1:** Iterative Estimation Algorithm for Rényi Entropy of Order 2 (Collision Entropy)

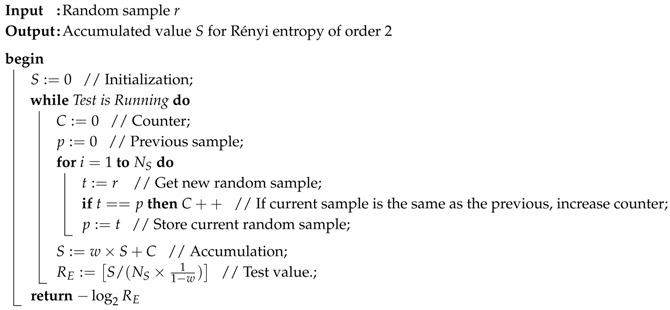

**Proposition** **2.** *For the stationary random source, after sufficiently large number of iterations, the test value in Algorithm 1 will converge to the Rényi entropy of order 2 (collision entropy).*

**Proof.**  For convenience, let us introduce indices to the counted value *C* and the accumulated value *S* in Algorithm 1 such as $C_{k}$ and $S_{k}$ where $1\leq k\leq N_{I}$. Then, the final accumulated value in Algorithm 1 is given as

$$\begin{array}{c} S_{N_{I}}=\sum_{k=1}^{N_{I}}w^{N_{I}-k}C_{k}. \end{array}$$

Suppose that the bias of random sample is stationary. That is, the bias level which can be represented as Pr(1) in the binary representation is fixed for several consecutive iterations, namely $N_{I}$ iterations. Then, we can substitute the counted values $C_{k}$ with the average of them, $\bar{C}$ where

(26) $$\begin{array}{c} \bar{C}=\frac{1}{N_{I}}\sum_{k=1}^{N_{I}}C_{k}. \end{array}$$

Then, the $S_{N_{I}}$ can be represented as

$$\begin{array}{c} S_{N_{I}}=\bar{C}\sum_{k=1}^{N_{I}}w^{N_{I}-k}=\bar{C}\times \frac{1-w^{N_{I}}}{1-w}. \end{array}$$

For a large integer $N_{I}$, we have $S_{N_{I}}≅\frac{\bar{C}}{1-w}$. That is, $R_{E}:=[S/(N_{S}\times \frac{1}{1-w})]$ in Algorithm 1 can be rewritten as

$$\begin{array}{c} R_{E}=\frac{S_{N_{I}}}{N_{S}\times \frac{1}{1-w}}≅\frac{\bar{C}\times \frac{1}{1-w}}{N_{S}\times \frac{1}{1-w}}=\frac{\bar{C}}{N_{S}}. \end{array}$$

That is, the output $R_{E}$ of Algorithm 1 is the average of counted values from the basic tests over the sample size $N_{S}$. Due to the time average in (26), the proposed algorithm can give us more stable estimated entropy values of the given random samples. ☐

The weight *w* will determine a trade-off between converging speed and reducing fluctuation, which will be shown in the next section. If *w* is close to 1, the estimated value shows less fluctuation at the cost of the sensitivity to the bias changes. In addition, if we choose $w=\frac{2^{m}-1}{2^{m}}$, then the multiplication in the final step corresponds to *m*-bit left shift. Moreover, $wS=\left(2^{m}S-S\right)/2^{m}$ in Algorithm 1 can be implemented using *m*-bit left shift, $\left\lceil \log_{2}S\right\rceil$-bit subtraction, and *m*-bit right shift.

## 4. Numerical Results

In this section, we present simulation results for the proposed entropy estimator with distinct sample sizes. Simulation results are presented in three ways. First, we present the estimation performance of two sample sizes for Rényi entropy of order 2. Second, we show estimation performance for Rényi entropy of order 1/2. Finally, we present the result of estimating Rényi entropy in an iterative manner.

### 4.1. Simulation for Rényi Entropy of Order 2

In this simulation, we use L=4, 6, and 8-bit blocks as a single input to the estimator. Therefore, the maximum entropies are also 4, 6 and 8-bit, respectively. For the simulation of the proposed estimator for the Rényi entropy of order 2, we choose two sample sizes; $K_{1}$ = 256,000 and $K_{2}$ = 10,240,000 which will be called the moderate sample size and the large sample size in the subsequent discussion, respectively. The moderate sample size is the same as the sample size specified in the entropy test for L=8 of AIS.31 for physical random number generators [5]. For the simulation, we generate 500 random sequences with two distinct lengths $K_{1}$ and $K_{2}$. Each random sequence has a specified bias, which is represented as probability of occurring 1, Pr(1), in a binary random sequence with a range from 0.001 to 0.5.

**Figure 1:**
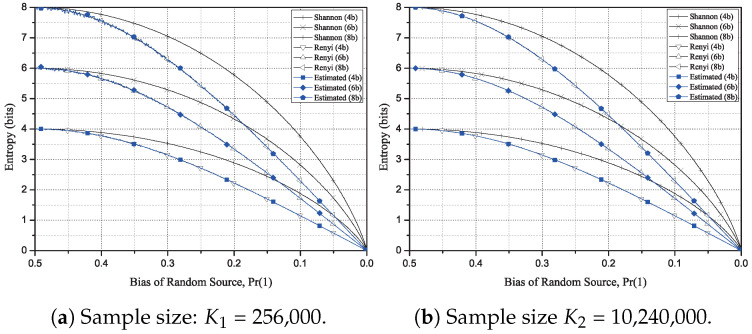
Entropy calculation and estimation for 4-, 6-, and 8-bit blocks of two sample sizes. The estimated Rényi entropy of order 2 closely follows the real entropy value for high statistical bias, while it fluctuates for low bias (Pr(1)→0.5).

Figure 1a shows the simulation results for the Rényi entropy of order 2 with the moderate sample size $K_{1}$ = 256,000. Notice that the test results are more accurate for high bias case (close to 0) than the low bias case (close to 0.5) clearly presented in Figure 1a for the sample size $K_{1}$. The simulation results for the Rényi entropy of order 2 with the large sample size $K_{2}$ = 10,240,000 are depicted in Figure 1b. In this figure, it is easy to see that the Rényi entropy of order 2 is less than or equal to the Shannon entropy as represented in (3). In addition, the test values of the proposed scheme are almost close to the Rényi entropy of order 2 as asserted in Proposition 1.

Let us evaluate the amount of fluctuation for a given sample size *K*. Denote the probability of occurring block *b* as $p_{b}$. The amount of the fluctuation of the test values can be represented using the standard deviation $\sigma_{U}$ of the number of occurrences of each block as in the following equation:

$$\begin{array}{c} f_{R}\left(s^{K}\right)=\log_{2}K-\log_{2}\left(m_{U}\pm k\sigma_{U}\right)=\log_{2}K-\log_{2}m_{U}-\log_{2}(1\pm \frac{k\sigma_{U}}{m_{U}}), \end{array}$$

where $m_{U}$ is the mean of the number of occurrences of each block and *k* is the number of standard deviations as which the test value is allowed to be away from the mean value. The mean and variance can be represented as

$$\begin{array}{cc} m_{U} & =\sum_{b\in F_{2}^{L}}K{\left[\mathrm{Pr}(b)\right]}^{2},\\ \sigma_{U} & =\sum_{b\in F_{2}^{L}}\mathrm{Pr}\left(b\right)\sqrt{K\mathrm{Pr}(b)(1-\mathrm{Pr}(b))}. \end{array}$$

Since the amount of the fluctuation is maximized at the no bias case (i.e., Pr(1)=0.5), we can write the test function at that case as follows:

$$\begin{array}{c} f_{R}\left(s^{K}\right)=\log_{2}\frac{1}{p_{b}}-\log_{2}(1\pm k\sqrt{\frac{1-p_{b}}{Kp_{b}}}). \end{array}$$

Then, the amount of fluctuations at no bias case for $K_{1}$ can be evaluated as in the following example.

**Example** **1.** *For example, suppose that K=256,000, L=8, k=2.58 (for 99% confidence), and the random sample has no bias. Then, $p_{b}=1/256$, $m_{U}=Kp_{b}=1000$, and $2.58\sigma_{U}=2.58\sqrt{Kp_{b}\left(1-p_{b}\right)}=81.42$. Therefore, we have the test function of the Rényi entropy of order 2 given as*

$$\begin{array}{c} 8-0.1129<f_{R}\left(s^{K}\right)<8+0.1225 \end{array}$$

*with 99% confidence.*

**Figure 2:**
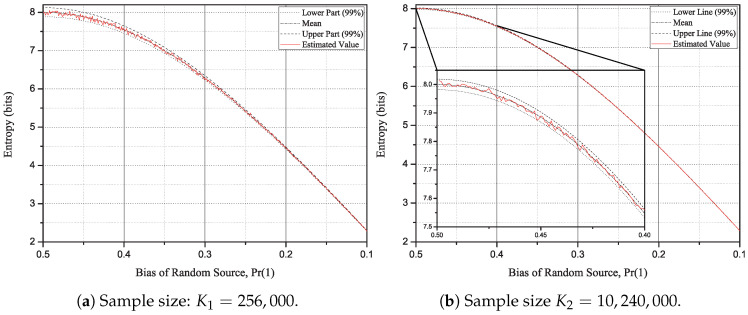
Deviation range of the test function for the given statistical bias and two sample sizes.

In Figure 2a, the center line (dashed-dot) is the mean value of test function. The upper (dot) and lower (dashed) lines correspond $2.58\sigma_{U}$ and $-2.58\sigma_{U}$ lines, respectively. That is, with 99% confidence, we can say that the test value of the Rényi entropy of order 2 will be between the upper and lower lines. In fact, the estimated value line (solid) is located between the upper and the lower lines in Figure 2a. For the Rényi entropy of order 2 with the large sample size, Figure 2b shows that the three lines are almost merged even in the no bias case. As we can check in the enlarged box on the left side of Figure 2b, the deviation from the real entropy value is small.

Note that, in the randomness test, we are more interested in checking whether the given sample is random or not, rather than in identifying the exact test value. Thus, it is enough that the test value is accurate within the around of the specified threshold. Therefore, we can find a suitable sample size according to the application and accuracy of the test. In particular, when the post-processed TRNGs are available, it is more important to detect a low entropy value because the post-processing method can reduce some statistical bias in the random samples [36].

### 4.2. Simulation for Rényi Entropy of Order 1/2

In this simulation, we use L=4, 6, and 8-bit blocks as a single input to the estimator of Rényi entropy of order 1/2. For the case of the proposed estimator for the Rényi entropy of order 1/2, we also choose two sample sizes; $K_{1}$ = 256,000 and $K_{3}$ = 1,024,000. For the simulation, we generate 500 random sequences with two distinct lengths $K_{1}$ and $K_{2}$. Each random sequence has a specified bias, which is represented as probability of occurring 1, Pr(1), in a binary random sequence with range from 0.001 to 0.5.

**Figure 3:**
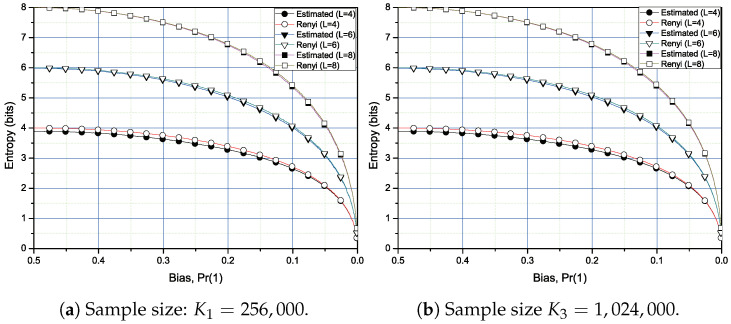
Rènyi entropy of order 1/2 calculation and estimation for 4-, 6-, and 8-bit blocks of two sample sizes. The first five values of exact g(k) are used. Remainder values are approximated using (21).

**Figure 4:**
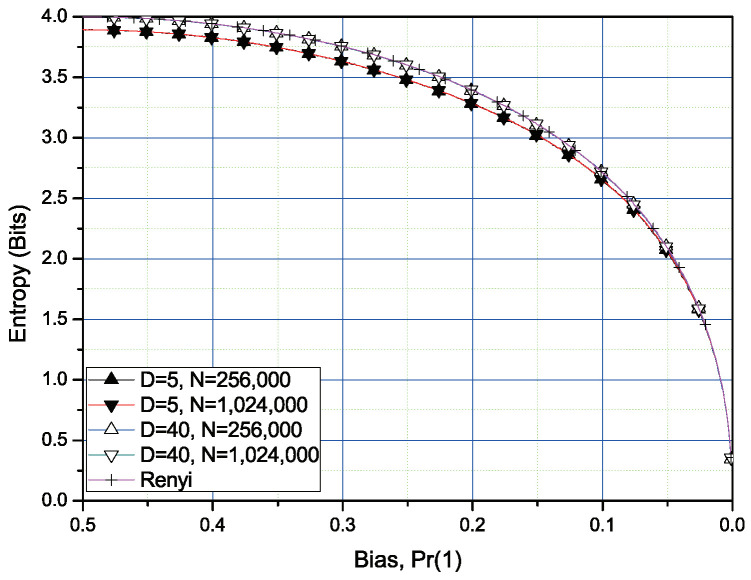
Entropy estimations the Rényi entropy of order 1/2 with the different number of uses of the exact parameters of g(k) for 4-bit blocks. of the sample size. *D* is the number of the exact values of parameters of g(k).

In Figure 3a,b, the simulation results for the Rényi entropy of order 1/2 with the moderate sample size $K_{1}$ = 256,000 and large sample size $K_{3}$ = 1,024,000. Note that, for L=8, the estimated entropy is almost matched with the actual values of the Rényi entropy of order 1/2 except for the bias range from 0.2 to 0.05. However, for L=6, the deviation of the estimated entropy from the exact Rényi entropy of order 1/2 is slightly greater than that of the case for L=8. For L=4, there exists the greater deviation between the estimated entropy values and the exact entropy values. This is because we only use the first five exact values of g(k) in (20) in the simulation. If we increase the number of uses of exact values of g(k) instead of approximated values in (21), we can improve the quality of the estimation as in Figure 4. In Figure 4, we compare the results obtained by the uses of the first five exact values and the first 40th exact values, respectively.

### 4.3. Simulation for Iterative Estimation Scheme of Rényi Entropy of Order 2

**Figure 5:**
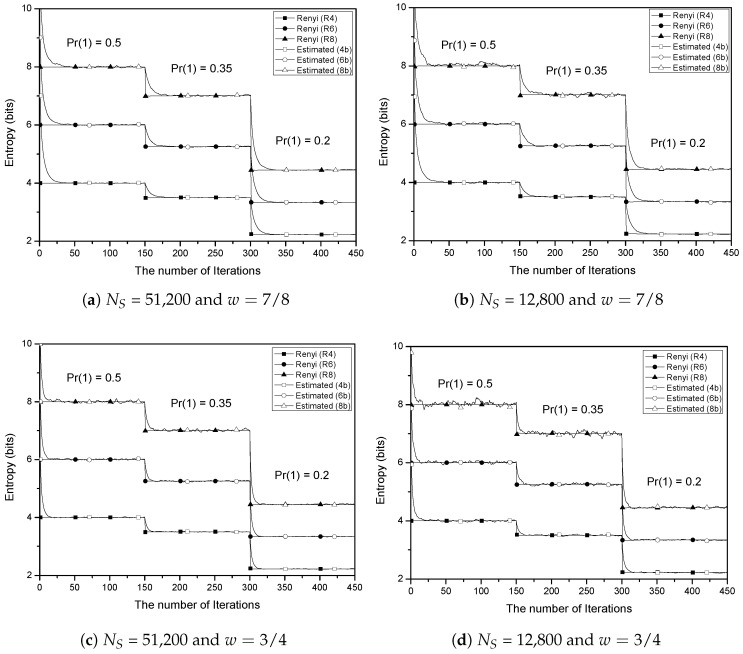
Iterative Rényi entropy estimation based on the basic test with two distinct sample sizes and weights. At the 150th and 300th iterations, the statistical bias is changed from Pr(1)=0.5 to Pr(1)=0.35, and to Pr(1)=0.2, respectively.

Finally, since increasing sample size usually involves tangible cost, collecting of a large number of random samples is not suitable for the constrained devices. In that case, we can apply the iterative estimation scheme presented in Section 3.4 with the smaller size of random sample instead of collecting large random samples for a single entropy estimation. Figure 5 shows the estimation results using Algorithm 1 with weight value 7/8 for the sample sizes 51,200 and 12,800, respectively. In each subfigure, three block sizes such as four, six, and eight bits are tested. In Figure 5, the first 150 iterations show the accumulated test results for the no bias case, i.e., the probability of one in the binary representation of random samples Pr(1)=0.5. Due to the initialization of Algorithm 1, the first few iterations show relatively big estimated value. For instance, if $N_{I}=1$, we have $S=C_{1}$ and $R_{E}=\frac{C}{8N_{S}}$. That is, the estimated value is increased by three bits such that $-\log_{2}R_{E}=3-\log_{2}\frac{C}{N_{S}}$. However, after about 25 iterations, the accumulated value converges to the Rényi entropy of order 2. Then, after the first 150 iterations, Pr(1) is suddenly changed from 0.5 to 0.35. In that situation, the accumulated test value smoothly converges to the new Rényi entropy value of order 2 for Pr(1)=0.35 within about 25 iterations again. Finally, after the first 300 iterations, the probability of one is abruptly changed again from 0.35 to 0.2. Similar to the previous bias change (0.5 → 0.35), the accumulated test value accordingly converges to new entropy value. Since the number of possible alphabets are exponentially increasing according to the block size *L*, for a given sample size $N_{S}$, the smaller block size (i.e., L=4) shows less fluctuating test results.

Figure 5c,d show the simulation results of Algorithm 1 with weight 3/4. The overall tendency is similar to the results in Figure 5a,b except that the converging speed becomes faster at the cost of higher fluctuations.

It is also interesting to check the convergence speed. How quickly the proposed method converges to the actual entropy value when the environment changes depends on the sample size $N_{S}$ and weight *w*. When we carefully observe Figure 5, it can be discovered that the larger the sample size and weight, the smaller the fluctuation. However, if the weight is large, the average value is reached at a slower rate. That is, the convergence speed is more related to weight than the sample size. However, it is not trivial to determine when the test value reached the real entropy of the random source.

## 5. Conclusions

In this paper, we proposed a new estimating method of the Rényi entropy of order α. After presenting the general representation of parameters of the proposed estimator, we investigate the simplified form of three particular orders, such as α→1, α=1/2, and α=2 in detail. It turned out that the proposed estimator of the Rényi entropy of order 2 which is the widely applicable order can be efficiently implemented by using counting and comparison logics for random samples. The main motivation for this research is to develop a lightweight randomness test method that does not require complex computations to be applicable to systems with limited computational environments such as in the various IoT (Internet of Things) devices. The proposed scheme has a useful and interesting property such that the higher statistical bias in the random sequences, the more accurate detection of that bias for moderate sample size. Because the detection of high bias cases is more critical for the TRNG evaluation, the proposed scheme is acceptable as an on-the-fly entropy estimator with a moderate sample size. However, for the accurate estimation over the wide range of biases, we should test a large amount of random samples. Therefore, we propose an iterative algorithm that continuously carries out the basic tests for the relatively short sample size and updates the accumulated test value. Although it is demonstrated that the proposed method can estimate Rényi entropy of order α, more research on accuracy and convergence speed of the proposed method is also required. We keep this problem as further work.

## Figures and Tables

**Table 1:** Values of the parameter g(k) of the proposed estimator for some α’s.

α∖k	1	2	3	4	5	6	7	8	9	10	⋯	*n*
$\frac{1}{2}$	1	$\frac{3}{2}$	$\frac{15}{8}$	$\frac{35}{16}$	$\frac{315}{128}$	$\frac{693}{256}$	$\frac{3003}{1024}$	$\frac{6435}{2048}$	$\frac{109395}{32768}$	$\frac{230945}{65536}$	⋯	$\frac{(2n-1)!}{4^{n-1}{\left(\left(n-1\right)!\right)}^{2}}$
α→1	1	1	1	1	1	1	1	1	1	1	⋯	1
2	1	0	0	0	0	0	0	0	0	0	⋯	(−1)n−11n−1
3	1	−1	0	0	0	0	0	0	0	0	⋯	(−1)n−12n−1
4	1	−2	1	0	0	0	0	0	0	0	⋯	(−1)n−13n−1
5	1	−3	3	−1	0	0	0	0	0	0	⋯	(−1)n−14n−1
6	1	−4	6	−4	1	0	0	0	0	0	⋯	(−1)n−15n−1
7	1	−5	10	−10	5	−1	0	0	0	0	⋯	(−1)n−16n−1
8	1	−6	15	−20	15	−6	1	0	0	0	⋯	(−1)n−17n−1
9	1	−7	21	−35	35	−21	7	−1	0	0	⋯	(−1)n−18n−1
10	1	−8	28	−56	70	−56	28	−8	1	0	⋯	(−1)n−19n−1

**Table 2:** Comparison of required number of operations for given *N* samples.

Method	Required Number of Operations
Maurer [8]	(N−1)L+(N−1)S+D
Coron [7]	(N−1)L+3(N−1)S+(3N+1)D
Proposed (α=2)	L+(N−1)S+D

*L*: logarithm, *S*: summation, and *D*: division.
